# Dapagliflozin-induced Late-onset Euglycemic Diabetic Ketoacidosis

**DOI:** 10.7759/cureus.6089

**Published:** 2019-11-07

**Authors:** Iqra Iqbal, Mohsin Hamid, Muhammad Atique Alam Khan, Aleesha Kainat, Shafaq Tariq

**Affiliations:** 1 Internal Medicine, Abington Hospital - Jefferson Health, Abington, USA; 2 Internal Medicine, King Edward Medical University, Lahore, PAK

**Keywords:** sglt-2 inhibitors, dapagliflozin, euglycemic dka

## Abstract

Sodium-glucose co-transporter-2 (SGLT2) inhibitors are a class of oral hypoglycemics that improve glycemic control by increasing the urinary excretion of glucose. They gained widespread popularity because they not only showed improved glycemic control but also had a favorable effect on weight loss, blood pressure, and cardiovascular mortality. One of their rare side effects is euglycemic diabetic ketoacidosis (eDKA) although the diagnosis is sometimes difficult to make due to near-normal glucose levels. We present a case of eDKA in a patient who presented with confusion, acute kidney injury (AKI), and metabolic acidosis after having an influenza-like illness with a minimally elevated blood glucose of 187 mg/dL. She had already stopped taking dapagliflozin (an SGLT-2 inhibitor) two weeks before the presentation. She was initially treated as sepsis and required hemodialysis. Later on, metabolic acidosis was attributed to eDKA from dapagliflozin, which resolved after the administration of intravenous insulin. Her eDKA developed while she had already stopped dapagliflozin two weeks ago, which makes this an interesting case finding. It is one of those rare cases where dapagliflozin led to a delayed complication of eDKA.

## Introduction

Since the discovery of the first oral hypoglycemic, i.e., a sulfonylurea in 1955, oral hypoglycemics have evolved as the first line of treatment for type II diabetes [1]. The search for a “perfect” oral hypoglycemic led to the discovery of multiple classes of drugs with the aim of not only improving glycemic control but also of having other beneficial effects such as weight loss, increase in insulin sensitivity, improvement in microvascular complications, and reduced cardiovascular mortality. Each class of oral hypoglycemic drugs showed some beneficial effects but, unfortunately, had some unusual adverse reactions as well. The newest oral hypoglycemic class of drugs introduced is sodium-glucose cotransporter-2 (SGLT2) inhibitors available since 2013. Although they had very promising initial results, the data regarding their long-term safety is scarce. We are presenting this case to highlight the rare adverse effects of acute kidney injury and delayed euglycemic diabetic ketoacidosis from dapagliflozin.

## Case presentation

A 75-year-old Caucasian female presented to the emergency room (ER) in January for a change in mental status and confusion after she was found wandering outside her home. The patient complained of generalized myalgias, nonproductive cough, and runny nose in the preceding few days for which she called her primary care physician and was given a script of oseltamivir, attributing the symptoms to influenza virus infection. Pertinent past medical history included hypertension, chronic kidney disease (CKD) stage III, with the baseline estimated glomerular filtration rate (eGFR) 45 milliequivalent/liter, and type II diabetes (DMT-2). Her medications included metformin, pioglitazone, amlodipine, atorvastatin, and ezetimibe. She used to live by herself and didn’t drink or smoke. Vitals in the ER were temperature: 93 F, pulse: 55/min, blood pressure: 96/54 mmHg, oxygen saturation: 98% on ambient air, and respiratory rate: 28/min.

**Figure 1:**
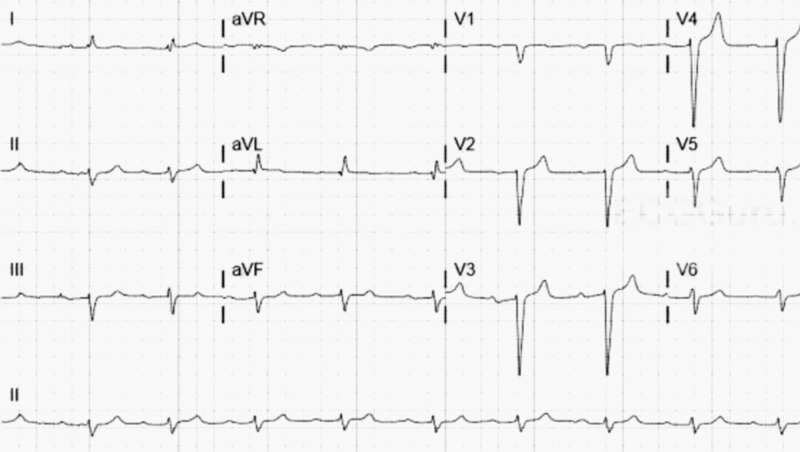
EKG on admission EKG: electrocardiogram

Physical examination showed that she was lethargic and oriented only to self, with dry mucosal membranes and cold, clammy skin. The neck was supple; extraocular movements were intact. Lungs were clear to auscultation. The rest of the examination, including the cardiovascular and gastrointestinal systems, were unremarkable. Pertinent laboratory evaluation, including complete metabolic profile (CMP) showed serum glucose of 187 mg/dL, creatinine: 11.5 mg/dL (baseline 1.8 mg/dl), sodium: 131 meq/L, potassium: 7.9 meq/L, bicarbonate: 5 meq/L, anion gap: 35, and glycosylated hemoglobin (HbA1c) of 6.2 mg/dL. Complete blood count (CBC) showed hemoglobin of 10 g/dL, platelets 370,000/uL, and white blood cells (WBC): 9.3 k/uL. The coagulation profile was normal. The lactic acid level was eight (8) meq/L. Venous blood gas analysis showed pH: 7.009, pCO2: 18.2 mmHg, and bicarbonate level: 5.1 mmol/L. Serum osmolarity was 312 mOsm/kg, with an osmolar anion gap of 12 mOsm/kg. Urinalysis showed glucose of 500 mg/dL, proteinuria of 30 mg/dL. Electrocardiogram (EKG) showed a first-degree heart block and broad QRS complex, as shown in Figure 1.

Computerized tomographic scan (CT) head and chest X-ray were unremarkable.

She was aggressively resuscitated with intravenous (IV) fluids. Hyperkalemia was treated with IV insulin, dextrose, calcium gluconate, sodium bicarbonate, and inhaled albuterol. Urgent hemodialysis was also arranged. Attributing her acute severe metabolic acidosis to influenza complicated by bacterial superinfection, she was started on broad-spectrum antibiotics and oseltamivir, and workup for acute renal failure, including antinuclear antibody (ANA), antineutrophilic cytoplasmic antibody (ANCA), complement levels, urine eosinophils, and renal ultrasound, was ordered. After hemodialysis (HD), electrolytes improved to a bicarbonate level of 18 mmol/L, potassium of 3.8 meq/L, and a pH of 7.433. She remained hemodynamically stable at this point. Repeat metabolic profile (BMP) fours hour after the completion of HD showed the bicarbonate level dropping down to 13 meq/L again. Endocrinology was consulted due to persistent metabolic acidosis. At this point, the patient recalled that she was also taking dapagliflozin (an SGLT-2 inhibitor) up to two weeks before the presentation when she stopped taking it due to her improving HbA1c.

Her serum beta-hydroxybutyrate result came back at 92.5 mg/dl. She was started on IV insulin and dextrose. Serum beta-hydroxybutyrate started improving and resolved entirely within 15 hours of beginning IV insulin, and bicarbonate improved to 22 meq/l. Later on, her urine output improved and electrolytes stabilized without further need for HD. Creatinine trended down and stabilized at 2.5 meq/L.

She resumed a regular diet, and beta-hydroxybutyrate remained within the normal range. Her blood sugar levels remained in the range of 110-150 mg/dl without hypoglycemic agents. Workup for acute renal failure came back negative. She was discharged to a rehabilitation center with the plan to establish her diabetic regimen, depending on blood sugar levels on an outpatient basis.

## Discussion

Euglycemic diabetic ketoacidosis (eDKA) is defined as the presence of metabolic acidosis (pH<7.3), positive urinary or serum ketones, serum bicarbonate <18 mmol/L, and blood glucose less than 200mg/dL; or in other words, it is DKA without hyperglycemia [2]. Munro first described eDKA in 1973 in people with type I diabetes who had decreased carbohydrate intake with the continued use of the same or increased amounts of insulin [3]. DKA is a medical emergency, and the mortality rate from DKA in the elderly above the age of 70 can be as high as 25% [4]. Historically, DKA has been mostly reported in type I diabetes and rarely in type II diabetes. The diagnosis of DKA entails the urgent initiation of IV fluids, insulin, aggressive potassium replacement, and management of the precipitating condition. Delay in the recognition or treatment of DKA can lead to severe complications such as hypokalemia, shock, acute respiratory distress syndrome (ARDS), and, possibly, even death.

Sodium-glucose cotransporter-2 inhibitors (SGLT-2 inhibitors) are the first class of oral hypoglycemics to lower serum glucose by acting on the kidneys and are glucouretic by their mechanism. SGLT-2 is a protein that is expressed in the proximal convoluted tubules (PCTs), responsible for the majority of the glucose absorption from the filtered load, and works independently of insulin. SGLT-2 inhibitors bind SGLT-receptors and decrease the absorption of glucose load to about 30%-50% and increase its excretion in urine [5]. Increased glucose load in the tubular lumen leads to osmotic diuresis and increased excretion of sodium along with it, i.e., natriuresis, helps improve the fluid balance in heart failure. Increased glucose delivery to the distal tubular cells leads to increased glucose absorption in exchange for uric acid, which has been hypothesized to cause a 5%-10% fall in the levels of uric acid in the blood and a subsequent fall in systemic and glomerular hypertension This uricosuria can also cause tubular injury [6]. The increased excretion of glucose and the associated calories results in a negative caloric balance, causing weight loss. Decreased serum glucose requires less insulin for normoglycemia and helps mitigate insulin resistance in type II diabetes. The mechanism of action of SGLT-2 doesn’t interfere with de novo glucose production and insulin secretion. Therefore, the risk of hypoglycemia doesn’t increase when used with other classes of oral hypoglycemics and insulin [7-8]. Nonspecific SGLT-2 inhibitors can also inhibit SGLT-1 receptors located in the bowel wall, resulting in reducing postprandial hyperglycemia [9].

SGLT-2 inhibitors, by their favorable extra-glycemic effects and insulin-independent mechanism of action, presented an attractive option for people with type I diabetes who, despite being compliant with the insulin regimen, suffer from extreme glucose swings, weight gain, hypertension, and other microvascular complications. It led to an exponential increase in the off-label use of SGLT-2 inhibitor usage in type I diabetics [10]. The most common adverse reactions with their use are female genital mycotic infections, urinary tract infections, and nasopharyngitis. In 2015, the FDA reported 73 cases of eDKA, and the European Medicines Agency (EMA) announced that it had received reports of 101 cases of eDKA with SGLT-2 inhibitors [11-12]. These warnings led to increased skepticism about this class of drugs, and a review done by Olga et al. showed that SGLT-2 inhibitors, amongst all antidiabetic medicines, have the highest rate of discontinuation, although the authors didn’t specify the reasons [10].

The primary mechanism in the eDKA is the decreased intake of carbohydrates. The pathophysiology of eDKA is almost similar to DKA except that ketoacidosis can occur in the presence of normoglycemia or minimally elevated glucose levels. SGLT-2 inhibitors can cause glucosuria of 50-100 g/day, comprising 17%-44% of the total daily carbohydrate intake and is associated with an asymptomatic increase in the β-hydroxybutyrate level of up to 12-20% [13-14].

Decreased serum glucose due to glucouretic effects of SGLT-2 inhibitors and reduced carbohydrate intake in times of stress leads to a decrease in insulin secretion. Low insulin levels, in turn, lead to increased glucagon secretion due to two mechanisms: 1) lack of inhibition of glucagon release from insulin and 2) inhibition of glucose uptake by alpha paracrine cells mediation by SGLT inhibition. Lower insulin to glucagon ratio and increased counter-regulatory hormones in times of stress lead to accelerated lipolysis in peripheral adipose tissue releasing free fatty acids that undergo beta-oxidation once inside hepatocytes to produce keto acids. Decreased insulin to glucagon ratio also leads to reduced glycogenolysis and increased gluconeogenesis, and the final serum glucose concentration being determined by endogenous glucose production and renal glucose clearance [15]. Therefore, one of the main differences between eDKA and DKA is the renal glucose clearance (ratio of glucosuria to serum glucose), which can be twice as high in eDKA as in DKA [16].

The plasma half-life of dapagliflozin is 13 hours, and it is 91% protein bound. It is metabolized mainly by the liver, and in negligible amounts by kidneys, i.e., <2%. It should be out of the system within three days, which is after six half-lives. Dapagliflozin should not be started if the estimated glomerular filtration rate (eGFR) is less than 60 mL/min/1.73 m². Elimination half-life gets prolonged by approximately three to six hours in chronic kidney disease and is not well-correlated with the degree of CKD. In our case, the patient developed eDKA two weeks after discontinuing dapagliflozin, which is unexpected compared to the published data of elimination half-life. A review done by Peters et al. suggested that the effect of SGLT-2 receptors might be longer than currently reported [17]. A case reported by Pujara also had persistent ketosis and glucosuria eight days after discontinuing dapagliflozin [18]. High protein binding or decreased eGFR might explain the prolonged effect, but no clear explanation was found in the literature. The clinical significance of the prolonged effect also lies in the perioperative settings, as the current recommendation is to suspend the drug three days before the surgery but if the impact of the drug is prolonged, it might put patients at risk of eDKA necessitating careful monitoring of the patient in the perioperative period.

In 2015, the FDA received reports of 101 cases of acute kidney injury (AKI) due to SGLT-2 inhibitors, which led to another warning against their use. Increased osmotic diuresis can lead to hyperosmolarity and dehydration. High glucose load in the proximal straight tubule can induce the gene responsible for an enzyme that can metabolize glucose into fructose, which can be converted later to uric acid and leads to the generation of chemokines, local inflammation, and tubular injury. However, it has been argued that in the cases reported to the FDA, some patients were already on diuretics, angiotensin-converting enzymes/angiotensin receptor blockers (ACE/ARB) inhibitors, or might have been hypotensive, which could have confounded the reporting [19].

In spite of these warnings, major organizations have recommended the continuation of the prescription of SGTL-2 for appropriate indications, as any other anti-diabetic medications hardly match the benefits provided by them. What needs to be stressed is the appropriate patient selection for SGLT-2 inhibitors and the increased awareness of their rare side effects to prevent any untoward outcomes. Patients should be started on SGLT-2 inhibitors for appropriate indications and screened carefully for any future adverse event. Dapagliflozin should not be started in patients with eGFR < 60 mL/min/1.73 m² and care should be taken if the patients are elderly and at high risk of hypotension, especially if they are already on diuretics and ACE/ARB inhibitors. Routine measurement of renal function should be done, and if eGFR < 60 mL/min/1.73 m², dapagliflozin should be discontinued. If the patient develops symptoms of nausea, decreased oral intake, they should be advised to measure serum ketones at home and present to ER if unable to tolerate oral intake. Low carbohydrate/Atkins diet and excessive alcohol use should be discouraged [20]. Dapagliflozin should be discontinued at least one week before anticipated surgery, and basal insulin should be used for glycemic needs.

Most importantly, more needs to be done to increase awareness of this rare but potentially fatal condition of eDKA amongst primary care/ER and critical care physicians. Patients should be carefully selected with appropriate indications for SGLT-2 inhibitors. eDKA is an underrecognized and possibly fatal adverse effect of SGLT-2 inhibitors; hence, measurements of serum ketones should be done whenever a patient taking SGTL-2 inhibitors gets sick. eDKA does not necessarily happen when the patient is currently taking medication; rather, it can also be a delayed side effect after the patient has stopped the medication. Routine analysis of kidney functions should be done, and dapagliflozin should be stopped once eGFR < 60 mL/min/1.73 m². Physicians should keep themselves informed of the latest updates on SGLT-2 inhibitors, as the prescribing information may change with the evolving medical knowledge.

## Conclusions

eDKA can present with an acute change in mental status, and the patient can be suffering from metabolic acidosis despite the relatively normal levels of blood sugar, which is similar to the presentation in our patient. eDKA is becoming a well-established side effect of SGLT2 drugs, but our case is unique in the way that it reports a delayed occurrence of eDKA when the patient was already off the medication. Another rare side effect of SGLT2 drugs is AKI, which presented concomitant to eDKA in this case. eDKA is also treated with the IV insulin infusion. AKI might need temporary dialysis sessions, but it usually resolves after the eDKA is treated, and, thus, our patient did not need long-erm dialysis. eDKA and AKI should be kept in mind when a patient presents with acidosis and is currently using or has used SGLT2 inhibitors.
